# Biological Effects of Ciliary Neurotrophic Factor on hMADS Adipocytes

**DOI:** 10.3389/fendo.2019.00768

**Published:** 2019-11-12

**Authors:** Jessica Perugini, Eleonora Di Mercurio, Giovanni Tossetta, Ilenia Severi, Federica Monaco, Marcella Reguzzoni, Marco Tomasetti, Christian Dani, Saverio Cinti, Antonio Giordano

**Affiliations:** ^1^Department of Experimental and Clinical Medicine, Marche Polytechnic University, Ancona, Italy; ^2^Department of Clinical and Molecular Sciences, Marche Polytechnic University, Ancona, Italy; ^3^Department of Surgical and Morphological Sciences, University of Insubria, Varese, Italy; ^4^Université Côte d'Azur, CNRS, INSERM, iBV, Faculté de Médecine, Nice, France; ^5^Center of Obesity, United Hospitals, Marche Polytechnic University, Ancona, Italy

**Keywords:** ciliary neurotrophic factor, adipocytes, JAK STAT pathway, lipolysis, mitochondria, inflammation, obesity, diabetes

## Abstract

Administration of ciliary neurotrophic factor (CNTF) to experimental animals exerts anti-obesity effects by acting on multiple targets. In white adipose tissue CNTF reduces lipid content, promotes fatty acid (FA) oxidation and improves insulin sensitivity. This study was performed to establish whether CNTF exerts similar effects on human white adipocytes. To this end, adipose differentiation was induced *in vitro* in human multipotent adipose-derived stem (hMADS) cells. CNTF receptor α (CNTFRα) expression was assessed in hMADS cells and adipocytes by qRT-PCR, Western blotting, and immunocytochemistry. After administration of human recombinant CNTF, signaling pathways and gene expression were evaluated by Western blotting and qRT-PCR. Glucose uptake was assessed by measuring 2-nitrobenzodeoxyglucose uptake with a fluorescence plate reader. Lastly, CNTF-induced anti-inflammatory responses were evaluated in hMADS adipocytes stressed with tumor necrosis factor α (TNFα) for 24 h. Results showed that CNTFRα protein expression was higher in undifferentiated hMADS cells than in hMADS adipocytes, where it was however clearly detectable. In hMADS adipocytes, 1 nM CNTF strongly activated the JAK-STAT3 (Janus kinase-signaling transducer and activator of transcription 3) pathway and acutely and transiently activated the AMPK (AMP-activated protein kinase) and AKT (protein kinase B) pathways. Acute CNTF treatment for 20 min significantly increased basal glucose uptake and was associated with increased AKT phosphorylation. Longer-term (24 and 48 h) treatment reduced the expression of lipogenic markers (FA synthase and sterol regulatory element-binding protein-1) and increased the expression of lipolytic [hormone-sensitive lipase (HSL) and adipose triglyceride lipase (ATGL)] and mitochondrial (peroxisome proliferator-activated receptor γ coactivator-1α and carnitine palmitoyltransferase 1) markers. In TNFα-treated hMADS adipocytes, CNTF significantly reduced the expression of monocyte chemoattractant protein 1 and TNFα-induced AKT inhibition. Collectively, these findings demonstrate for the first time that CNTF plays a role also in human adipocytes, driving their metabolism toward a less lipid-storing and more energy-consuming phenotype.

## Introduction

Ciliary neurotrophic factor (CNTF) was originally discovered as a neurotrophic factor contained in extracts of chick intraocular tissue, where it was capable of promoting the survival of ciliary ganglion neurons during a critical period of embryonic development (1). It was subsequently purified and cloned also in mammals where, in addition to other trophic actions, it was found to support the survival of brain motor neurons (2). These observations led to test human recombinant CNTF in amyotrophic lateral sclerosis patients (3, 4). Although its administration did not prove successful in slowing disease progression, it unexpectedly induced anorexia and weight loss. Importantly, the weight-reducing effect of exogenous CNTF was confirmed in leptin-resistant obese patients treated with subcutaneous Axokine, a human CNTF with enhanced specificity and potency (5). These findings stimulated considerable interest in the metabolic role of CNTF and highlighted the possibility of using this peptide, or its analogs, to treat human obesity and associated diseases.

Studies of animal models have consistently demonstrated that exogenous CNTF not only reduces food intake, by acting on hypothalamic (6–8) and brainstem (9) feeding centers, but also improves obesity-associated hyperglycemia, hyperinsulinemia, and hyperlipidemia by exerting metabolic effects through actions on peripheral organs (6, 10). In skeletal muscle, exogenous CNTF increases fatty acid (FA) oxidation, reduces insulin resistance and promotes muscle growth (11–13). In the liver of *db/db* obese mice (14) and of obese rats fed a high-fat diet (15) it reduces hepatic steatosis and enhances insulin responsiveness. Finally, in mice with alloxan-induced (16) and streptozotocin-induced (17) diabetes it protects pancreatic islet cells from cytokine-induced apoptosis, it increases β cell mass and reduces insulin clearance.

CNTF also exerts important effects on adipose tissue. Indeed, in mice weight loss due to CNTF hypersecretion by genetically modified implanted glioma cells results in fast and preferential loss of fat tissue (18). In obese patients the severity of dysfunctional adipocyte metabolism, adipokine dysregulation, and chronic subclinical inflammation determines the frequency and severity of a number of comorbid disorders including insulin resistance, dyslipidemia, hypertension, and cardiovascular disease (19–21). In cultured brown adipocytes CNTF enhances β_3_-adrenergic induction of mitochondrial uncoupling protein 1 (UCP1) (22), whereas in brown fat from normal and obese mice it upregulates UCP1 (23), potentially promoting non-shivering thermogenesis-dependent energy expenditure. Finally, it reduces lipogenesis and promotes mitochondrial biogenesis, FA oxidation and insulin sensitivity in mouse 3T3-L1 adipose cells and mouse white fat explants (24, 25).

Given the remarkable effects of CNTF on rodent white adipose tissue and its potential to treat human obesity, we performed a series of experiments to characterize the signaling systems, transcriptional changes, glucose uptake and inflammatory responses modulated by acute and/or long-term CNTF treatment using cultured adipose cells differentiated from human multipotent adipose tissue-derived stem (hMADS) cells (26, 27). Under proper conditions, these cells can differentiate into functional adipocytes and express the typical genetic and metabolic signatures of human white adipocytes, thus providing the best available *in vitro* model of human adipose cells. Collectively, our results show that CNTF exerts anti-obesity and anti-inflammatory effects also on human adipocytes.

## Materials and Methods

### Cell Culture and Treatments

The cell culture media, fetal bovine serum, buffers, and trypsin were from Pan-Biotech GmbH (Aidenbach, Germany); the cell culture reagents, including Oil Red O, curcumin, and insulin, were from Sigma-Aldrich (Milan, Italy). Human recombinant CNTF, human recombinant fibroblast growth factor (hFGF)-2 and human recombinant tumor necrosis factor α (TNFα) were purchased from PeproTech (London, UK). hMADS cells were cultured as previously described (26–28). In brief, hMADS cells grown in low-glucose (1 g/l) proliferation medium [Dulbecco's modified Eagle's medium (DMEM)] supplemented with 10% fetal bovine serum and 2.5 ng/ml hFGF-2 were used between the 16th and the 19th passage. To induce adipose differentiation, they were seeded in proliferation medium on multi-well plates at a density of 4,500 cells/cm^2^. When they reached confluence hFGF-2 was not replaced. The next day (day 0), cells were incubated in adipogenic medium (serum-free proliferation medium/Ham's F-12 medium) containing 10 μg/ml transferrin, 5 μg/ml insulin, 0.2 nM triiodothyronine, 100 μM 3-isobutyl-1-methylxanthine (IBMX), 1 μM dexamethasone, and 100 nM rosiglitazone. Dexamethasone and IBMX were not replaced from day 3 and rosiglitazone from day 9. Cell lipid content was assessed at different time points by Oil Red O staining (29). Treatments and biological assays were carried out on differentiated hMADS adipocytes from day 12 to 15. Treatment duration and the concentrations of CNTF, curcumin, TNFα, and insulin are reported in the Results section.

### qRT-PCR

Total RNA was extracted with TRIZOL reagent (Invitrogen, Carlsbad, CA, USA), purified, digested with ribonuclease-free deoxyribonuclease and concentrated using RNeasy Micro kit (Qiagen, Milan, Italy) according to the manufacturer's instructions. For determination of mRNA levels, 1 μg RNA was reverse-transcribed with the High-Capacity cDNA RT Kit with RNase Inhibitor (Applied BioSystems, Foster City, CA, USA) in a total volume of 20 μl. qRT-PCR was performed using TaqMan Gene Expression Assays and Master Mix TaqMan (Applied BioSystems). All probes (Table 1A) were from Applied BioSystems. Reactions were carried out in a Step One Plus Real Time PCR system (Applied BioSystems) using 50 ng RNA in a final reaction volume of 10 μl. The thermal cycle protocol included: initial incubation at 95°C for 10 min followed by 40 cycles of 95°C for 15 s and 60°C for 20 s. A control reaction without reverse transcriptase in the amplification mixture was performed for each sample to rule out genomic contamination. All samples were run in duplicate. Samples not containing the template were included as negative controls in all experiments. TATA box-binding protein (TBP) was used as an endogenous control to normalize gene expression. Relative mRNA expression was determined by the ΔCt method (2^−ΔCt^).

**Table 1A:** Taqman probes all from Applied Biosystems #4453320.

**Target gene**	**Assay ID**
Adiponectin	Hs00605917_m1
ATGL	Hs00386101_m1
CNTFRα	Hs00181798_m1
CPT1	Hs00912671_m1
FABP4	Hs01086177_m1
FAS	Hs01005622_m1
HSL	Hs00943410_m1
IL-1β	Hs01555410_m1
IL-6	Hs00174131_m1
PAI1	Hs01126604_m1
MCP1	Hs00234140_m1
Perilipin	Hs00160173_m1
PGC-1α	Hs01016719_m1
PPARγ	Hs01115513_m1
SREBP-1	Hs04984975_m1
SOCS3	Hs02330328_s1
TBP	Hs00427620_m1

### Western Blotting

hMADS cell lysates were obtained by using lysis buffer containing 50 mM Tris–HCl (pH 7.4), 1% NP-40, 1 mM EDTA, 150 mM NaCl, 1 mM sodium orthovanadate, 0.5% sodium deoxycholate, 0.1% SDS, 2 mM phenylmethylsulfonylfluoride, and 50 mg/ml aprotinin. Samples were centrifuged, and protein concentrations were determined by the Bradford Protein Assay (Bio-Rad Laboratories, Segrate, Italy). Proteins were separated by SDS-PAGE then transferred to a nitrocellulose membrane using the Trans-Blot Turbo^TM^ Transfer system (Bio-Rad). To check loading and transfer efficiency, membranes were visualized with Ponceau S solution (Santa Cruz Biotechnology, Santa Cruz, CA, USA). Membranes were then blocked for 1 h at room temperature (RT) in TBS-Tween-20 (50 mM Tris-HCL [pH 7.6], 200 mM NaCl and 0.1% Tween-20) containing 5% non-fat dried milk and subsequently incubated overnight at 4°C with the primary antibody (Table 1B). After washing in TBS-Tween-20 and incubation with the secondary antibody for 1 h at RT (Table 1C), bands were visualized with the Chemidoc Imaging system using the Clarity™ Western ECL chemiluminescent substrate (all from Bio-Rad). Quantitation of immunoreactive bands was performed using the Bio-Rad Image Lab software. Where appropriate, membranes were stripped, washed and re-probed for total protein content.

**Table 1B:** Primary antibodies.

**Antibodies**	Host^*^	**Dilution**	**Source**
AKT	R	1:1,000 (WB)	Cell Signaling Technology/9272
AMPKα	M	1:100 (WB)	Santa Cruz Biotechnology/sc-74461
ATGL	M	1:100 (WB)	Santa Cruz Biotechnology/sc-365278
β-Actin	M	1:200 (WB)	Santa Cruz Biotechnology/sc-477778
β-Tubulin	M	1:800 (WB)	Santa Cruz Biotechnology/sc-5274
CNTFRα	R	1:250 (WB)	Thermo Fischer Scientific/PA5-45053
CNTFRα	M	1: 50 (IF)	Abcam/ab89333
CPT1	M	1:50 (WB)	Santa Cruz Biotechnology/sc-393070
FAS	M	1:500 (WB)	Santa Cruz Biotechnology/sc-48357
pAKT (Ser473)	R	1:2,000 (WB)	Cell Signaling Technology/4060
pAMPKα (Thr172)	R	1:1,000 (WB)	Cell Signaling Technology /2535
PGC-1α	M	1:100 (WB)	Santa Cruz Biotechnology/sc-517380
pSTAT3 (Tyr705)	M	1:2,000 (WB)	Cell Signaling Technology/4113
Perilipin	R	1:100 (IF)	Abcam/ab3526
SOCS3	M	1:200 (WB)	Santa Cruz Biotechnology/sc-73043
STAT3	R	1:1,000 (WB)	Santa Cruz Biotechnology/9132

* *M, mouse; R, rabbit; WB, western blotting; IF, Immunofluorescence*.

**Table 1C:** Secondary antibodies.

**Conjugated to**	React^*^	**Dilution**	**Source**	**ID**
Peroxidase	M	1:5,000	Jackson ImmunoResearch	715-036-150
Peroxidase	R	1:1,000	Vector Laboratories	PI-1000
Fluorophore	M	1:100	Invitrogen	A31570
Fluorophore	R	1:100	Invitrogen	A21206

* *M, mouse; R, rabbit*.

### Immunofluorescence and Confocal Microscopy

For these experiments, hMADS adipocytes were differentiated in 4-well Lab-Tek Chamber Slides (Nalge Nunc International, Naperville, IL, USA), washed with phosphate buffered saline (PBS) pH 7.4, fixed with 4% paraformaldehyde in PBS for 20 min at RT, and permeabilized with PBS/0.1% Triton X-100 for 10 min. After three washes in PBS, they were blocked with 10% normal donkey serum (Jackson Immuno Research, West Grove, PA, USA) in PBS and incubated with a mixture of two primary antibodies (Table 1B) overnight at 4°C. The next day, they were thoroughly rinsed with PBS and incubated in a cocktail of fluorophore-linked secondary antibodies (Table 1C) for 1 h at RT. After washing with PBS, nuclear staining was performed with TO-PRO™-3 Iodide (Invitrogen). Finally, cells were air-dried and cover-slipped using Vectashield mounting medium (Vector Laboratories, Burlingame, CA, USA) and viewed under a motorized Leica DM6000 microscope at different magnifications. Fluorescence was detected with a Leica TCS-SL spectral confocal microscope (Wetzlar, Germany) equipped with an Argon and He/Ne mixed gas laser. Fluorophores were excited with the 488, 543, and 649 nm lines and imaged separately. Images (1,024 × 1,024 pixels) were obtained sequentially from two channels using a confocal pinhole of 1.1200 and stored as TIFF files. When the primary antibody was omitted staining was never observed.

### Lipolysis

Lipolysis was assessed through quantification of free FA (FFA) release into the culture media by using the Free Fatty Acid Quantification Assay Kit (# ab65341, Abcam, Cambridge, UK) that detects longer chain FFAs by an enzyme-based method. Absorbance was measured using a microplate spectrophotometer (Infinite F200 PRO plate reader) at 570 nm. IBMX, a non-selective phosphodiesterase inhibitor which raises intracellular cAMP and stimulates adipocyte lipolysis, was used as a positive control.

### Glucose Uptake

For these experiments, hMADS adipocytes were differentiated in 96-well black-bottom plates (Corning, Sigma-Aldrich Chemie, Milano, Italy) and incubated in low-glucose DMEM with 1 nM CNTF for different periods of time (see Results). Next, adipocytes were incubated with or without 100 nM insulin in Krebs-Ringer phosphate buffer (pH 7.4) at 37°C for 10 min. They were then treated with 2-nitrobenzodeoxyglucose (2-NBDG, 50 μM) for 60 min. Fluorescence intensity was evaluated at 550/590 nm using an Infinite F200 PRO plate reader (Tecan, Mannedorf, Switzerland).

### Statistical Analysis

All experiments were performed in triplicate. Data are reported as mean ± standard error of the mean (SEM). Comparisons between and among groups were performed, respectively, with Student's *t*-test and one-way analysis of variance (ANOVA). A *p* < 0.05 was considered significant. All statistical analyses were performed with GraphPad Prism 6 software.

## Results

### hMADS Adipocytes Express CNTFRα

**Figure 1:**
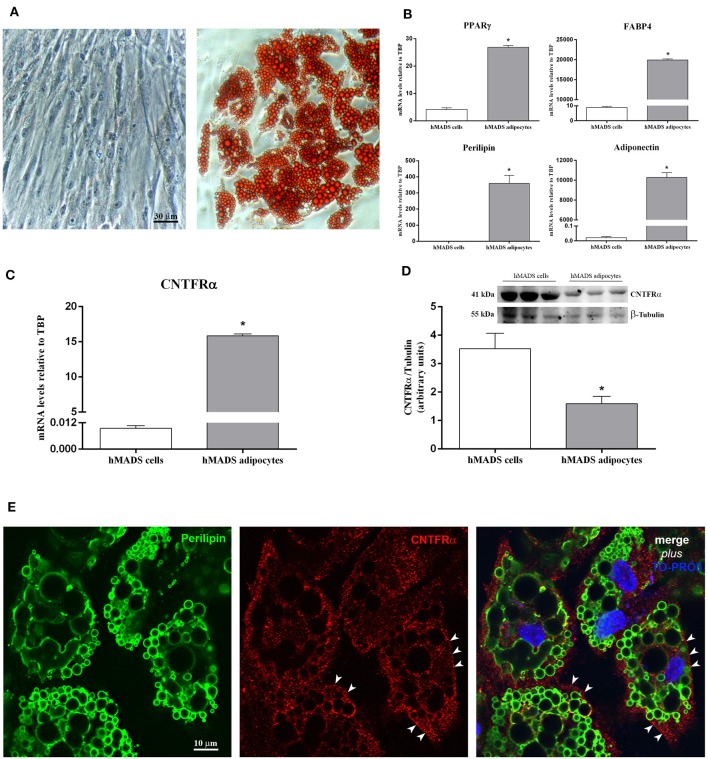
CNTFRα expression in undifferentiated cells and differentiated hMADS adipocytes. **(A)** Representative pictures from Oil Red O-stained confluent hMADS cells (day 0, left panel) and adipocytes (day 13, right panel). Data were analyzed using Student's *t*-test. **(B)** qRT-PCR analysis of PPARγ, FABP4, perilipin, and adiponectin expression in hMADS cells (white bars) and adipocytes (gray bars). Data (*n* = 3) are mean ± SEM, *p < 0.05 compared with hMADS cells. Data were analyzed using Student's *t*-test. qRT-PCR **(C)** and representative immunoblot and quantification **(D)** of CNTFRα expression in hMADS cells (white bars) and adipocytes (gray bars). Data (*n* = 3) are mean ± SEM, *p < 0.05 compared with hMADS cells. Data were analyzed using Student's *t*-test. **(E)** hMADS adipocytes: double immunostaining and confocal microscopy images showing perilipin staining (left, green) around lipid droplets and CNTFRα staining (middle, red) in the cytoplasm and on some tracts of the plasma membrane (arrowheads).

By light microscopy, Oil Red O staining performed at various stages of adipose differentiation demonstrated the well-known morphological progression of hMADS cells from fibroblast-like cells devoid of lipids (Figure 1A, left) to roundish cells with a multilocular lipid content, typical of fully differentiated hMADS adipocytes (Figure 1A, right). Between days 12 and 15 of differentiation, about 80% of cells showed an abundant multilocular lipid content. By qRT-PCR, hMADS adipocytes (unlike their undifferentiated counterparts) expressed the typical markers of mature white adipocytes, including peroxisome proliferator-activated receptor γ (PPARγ), fatty acid-binding protein 4 (FABP4), perilipin, and adiponectin (Figure 1B). CNTF receptor α (CNTFRα) mRNA was barely detectable in undifferentiated hMADS cells but it was abundantly expressed in hMADS adipocytes (Figure 1C). In striking contrast, Western blot analysis of protein extracts showed a greater expression of CNTFRα protein in undifferentiated cells than in hMADS adipocytes (Figure 1D). The mismatch between gene and protein expression suggests that during adipose differentiation CNTFRα protein synthesis progressively declined; in contrast, a substantial amount of CNTFRα mRNA was still detectable, possibly providing for “on-demand” mRNA translation in response to as yet uncharacterized stimuli. Notably, a progressive reduction in CNTFRα protein expression has been described in mouse 3T3-L1 cells undergoing adipose differentiation (24). We sought confirmation that differentiated hMADS adipocytes expressed CNTFRα protein by performing immunocytochemical experiments. Immunofluorescence and confocal microscopy analysis of hMADS adipocytes double-stained for perilipin and CNTFRα showed specific CNTFRα staining in the cytoplasm and on some tracts of the plasma membrane (Figure 1E). Collectively, these data demonstrate that hMADS adipocytes express CNTFRα, thus representing a suitable model to study the cellular effects of CNTF on human white adipocytes.

### CNTF Strongly Activates the JAK-STAT3 Pathway in hMADS Adipocytes

**Figure 2:**
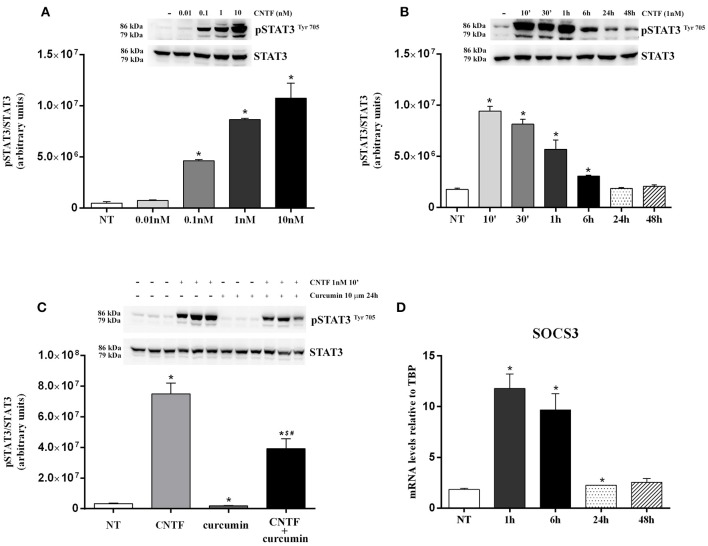
JAK-STAT3 pathway activation in hMADS adipocytes by CNTF. **(A)** Representative immunoblot and quantification of the dose-dependent increase in 705-tyrosine STAT3 phosphorylation in hMADS adipocytes treated with increasing concentrations of human recombinant CNTF for 10 min. Data (*n* = 3) are mean ± SEM, *p < 0.05 compared with untreated cells (NT). Data were analyzed using one-way ANOVA. **(B)** Representative immunoblot and quantification of the time-dependent STAT3 phosphorylation in hMADS adipocytes treated with 1 nM CNTF. Data (*n* = 3) are mean ± SEM, *p < 0.05 compared with untreated cells (NT). Data were analyzed using one-way ANOVA. **(C)** Representative immunoblot and quantification of STAT3 phosphorylation as a response to 1 nM CNTF treatment for 10 min, detected in hMADS adipocytes pretreated with 10 μM curcumin for 24 h. Data (*n* = 3) are mean ± SEM, *p < 0.05 compared with untreated cells (NT), $*p* < 0.05 compared with cells treated with curcumin, #*p* < 0.05 compared with cells treated with CNTF. Data were analyzed using one-way ANOVA. **(D)** Time-dependent SOCS3 mRNA induction in hMADS adipocytes by 1 nM CNTF treatment. Data (*n* = 3) are mean ± SEM, *p < 0.05 compared with untreated cells (NT). Data were analyzed using one-way ANOVA.

We first investigated which adipocyte-relevant signaling systems are activated by CNTF in differentiated hMADS adipocytes. The Janus kinase (JAK)-signaling transducer and activator of transcription 3 (STAT3) pathway regulates key white adipocyte functions (30), and the severity of its deregulation in obesity correlates with important pathological outcomes, including leptin and insulin resistance (31). Interestingly, the JAK-STAT3 pathway is the main transduction system coupled to CNTFRα activation (32, 33). Acute treatment of hMADS adipocytes with human recombinant CNTF for 10 min resulted in a dose-dependent increase in STAT3 phosphorylation at tyrosine 705 that was already significant at 0.1 nM CNTF and became progressively stronger at 1 and 10 nM CNTF (Figure 2A). Based on these findings and on data from CNTF-treated mouse 3T3-L1 adipocytes (24, 25), subsequent experiments were performed using 1 nM CNTF. To assess time dependence, JAK-STAT3 pathway activation was evaluated in hMADS adipocytes treated with CNTF for periods ranging from 10 min to 48 h. The results demonstrated that STAT3 phosphorylation was strongest after acute treatment (10 min) and that the longer the treatment the weaker the effect; indeed, in cells treated for 24 or 48 h the difference between treated and untreated cells was not significant (Figure 2B). Collectively, these experiments showed that 1 nM CNTF administered for 10 min induced a robust activation of JAK-STAT3 signaling in hMADS adipocytes. Notably, the activation was blunted by pretreatment with 10 μM curcumin, a widely used JAK-STAT3 inhibitor, for 24 h (Figure 2C) (34). Cellular activation of the JAK-STAT pathway induces translocation of phosphorylated STAT dimers to the nucleus, to regulate the transcription of target genes. Importantly, STAT transcription activity also stimulates the expression of the suppressors of cytokine signaling (SOCSs), which by acting as negative feedback signals further inhibit JAK-STAT phosphorylation and prevent negative cellular effects due to overactivation of the JAK-STAT pathway (35). To obtain a conclusive demonstration of the activation of the JAK-STAT3 pathway by CNTF and of its transcription potential, SOCS3 mRNA expression was evaluated in hMADS adipocytes treated with CNTF for different periods of time. We found that CNTF treatment for 1 and 6 h induced a strong increase in SOCS3 mRNA; SOCS3 transcription was still slightly upregulated at 24 h, whereas after 48 h of treatment its expression had reverted to baseline (Figure 2D).

### CNTF Transiently Activates the AMPK and AKT Pathways in hMADS Adipocytes

**Figure 3:**
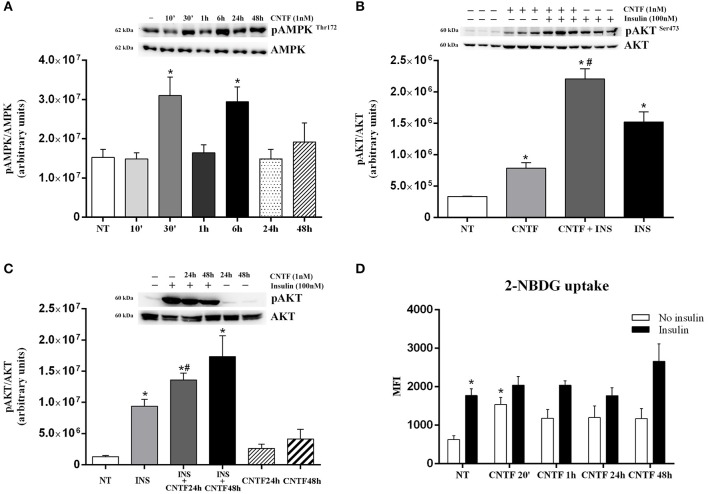
AMPK and AKT pathway activation in hMADS adipocytes by CNTF. **(A)** Representative immunoblot and quantification of time-dependent 172-threonine AMPK phosphorylation in hMADS adipocytes treated with 1 nM CNTF. Data (*n* = 3) are mean ± SEM, *p < 0.05 compared with untreated cells (NT). Data were analyzed using one-way ANOVA. **(B)** Representative immunoblot and quantification of 473-serine AKT phosphorylation in hMADS adipocytes treated with 1 nM CNTF for 20 min (CNTF); with CNTF and 100 nM insulin for 20 min (INS); and with insulin alone. Data (*n* = 3) are mean ± SEM, *p < 0.05 compared with untreated cells (NT), #*p* < 0.05 compared with cells treated with insulin alone. Data were analyzed using one-way ANOVA. **(C)** Representative immunoblot and quantification of 473-serine AKT phosphorylation detected in hMADS adipocytes treated with 100 nM insulin for 20 min (INS); with 1 nM CNTF for 24 h (CNTF 24 h + INS), or 48 h (CNTF 48 h + INS); only with CNTF for 24 (CNTF 24 h) or 48 h (CNTF 48 h). Data (*n* = 3) are mean ± SEM, *p < 0.05 compared with untreated cells (NT). Data were analyzed using one-way ANOVA. **(D)** 2-NBDG uptake, expressed as mean fluorescent intensity (MFI), detected in hMADS adipocytes treated with 1 nM CNTF alone applied for different periods (white bars) or else with 100 nM insulin for 10 min (black bars). Data (*n* = 3) are from three independent experiments and are mean ± SEM, *p < 0.05 compared with untreated cells (NT). Data were analyzed using Student's *t*-test.

In white adipocytes, the AMP-activated protein kinase (AMPK) pathway inhibits FA, cholesterol, and triglyceride synthesis and activates FA uptake and oxidation (36). To assess whether CNTF affects AMPK signaling in hMADS adipocytes, we measured 172-threonine phosphorylation of the α subunit of the AMPK complex, which is required for AMPK activation (37). Time course experiments showed significantly increased AMPK phosphorylation at 30 min and 6 h, whereas CNTF treatment for 10 min and for 1, 24, and 48 h induced no effect (Figure 3A). Even though time dependence was less linear than with the JAK-STAT3 pathway, these data suggest that CNTF acutely and transiently modulates the AMPK pathway in hMADS adipocytes. Notably, treatment with 10 ng/ml Axokine for 45 min has been reported to activate AMPK signaling in 3T3-L1 cultured adipocytes (25).

Next, we assessed the activation of the protein kinase B (AKT) pathway, which in adipocytes is strongly involved in basal and insulin-dependent glucose entry (38). Time dependence experiments showed that CNTF induced variable and non-significant 473 serine AKT phosphorylation in hMADS adipocytes, except for a significant peak at 20 min (data not shown). To confirm this acute effect of CNTF on AKT signaling, hMADS adipocytes were also treated for 20 min with 100 nM insulin, alone and combined with CNTF. As expected, AKT phosphorylation was induced by CNTF and, to an even greater extent, by insulin; interestingly, however, the combined treatment induced a significantly higher activation than insulin alone (Figure 3B), suggesting that in this condition CNTF enhanced insulin responsiveness in hMADS adipocytes. Furthermore, a similar, albeit not statistically significant effect, was seen in hMADS adipocytes pretreated with CNTF for 24 or 48 h and then treated with insulin for 20 min (Figure 3C). Since by modulating the AKT pathway CNTF could promote glucose entry into hMADS adipocytes (38), we measured 2-NBDG uptake by hMADS adipocytes treated with CNTF for different periods of time with and without insulin. The results showed that CNTF induced a slight increase in basal glucose uptake at all time points, although the difference was significant only after 20 min (Figure 3D). Insulin (100 nM) added for 10 min to CNTF-treated cells before 2-NBDG incubation induced a variable and non-significant increase in glucose uptake at all time points compared with the cells treated with insulin alone (Figure 3D).

### CNTF-induced Transcriptional Changes in hMADS Adipocytes

**Figure 4:**
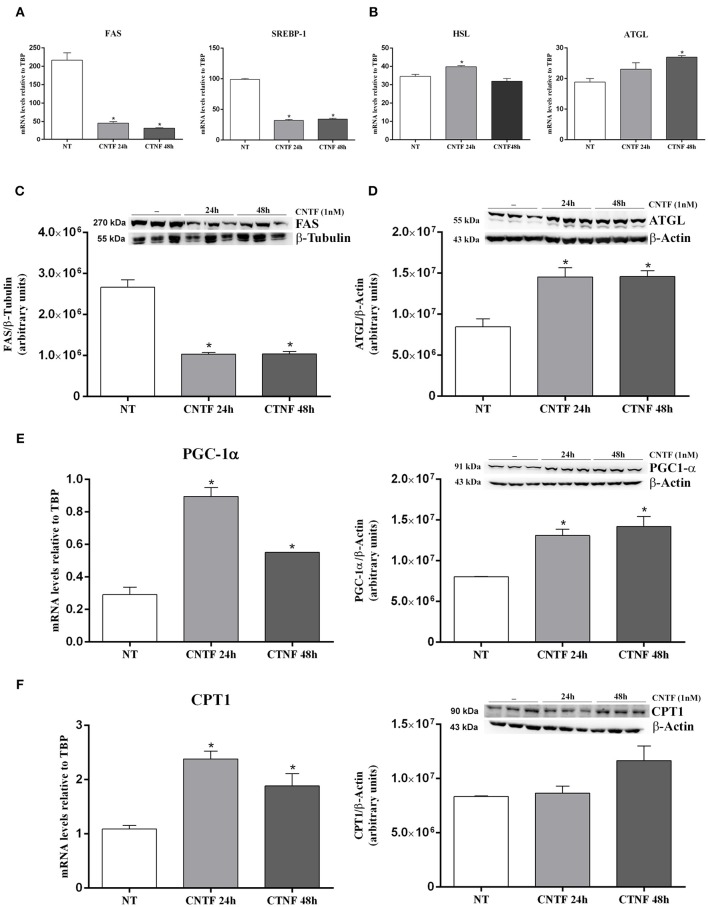
Transcriptional changes induced by long-term CNTF treatment in hMADS adipocytes. mRNA levels of SREBP-1 and FAS lipogenic markers **(A)** and HSL and ATGL lipolytic enzymes **(B)** in hMADS adipocytes treated with 1 nM CNTF for 24 or 48 h. Data (*n* = 3) are mean ± SEM, *p < 0.05 compared with untreated cells (NT). Data were analyzed using one-way ANOVA. Representative immunoblots and quantitative analyses of FAS **(C)** and ATGL **(D)** protein expression in hMADS adipocytes treated with 1 nM CNTF for 24 or 48 h. Data (*n* = 3) are mean ± SEM, *p < 0.05 compared with untreated cells (NT). Data were analyzed using Student's *t*-test. **(E)** PGC-1α mRNA and representative immunoblot and protein quantification in hMADS adipocytes treated with 1 nM CNTF for 24 or 48 h. Data (*n* = 3) are mean ± SEM, *p < 0.05 compared with untreated cells (NT). Data were analyzed using one-way ANOVA. **(F)** CPT1 mRNA and representative immunoblot and protein quantification in hMADS adipocytes treated with 1 nM CNTF for 24 or 48 h. Data (*n* = 3) are mean ± SEM, *p < 0.05 compared with untreated cells (NT). Data were analyzed using one-way ANOVA.

White adipocytes store large amounts of triglycerides that derive from FA and glycerol 3-phosphate esterification. FA are obtained from circulating lipoproteins or are *de novo* synthesized by FA synthase (FAS) from non-lipid substrates such as glucose. In mature fat cells, FAS gene expression is strictly regulated by sterol regulatory element-binding protein (SREBP)-1, a transcription factor promoting conversion of carbohydrates into lipids in both fat (39) and liver (40). During fasting or prolonged physical exercise, triglycerides from the lipid droplets are hydrolyzed by hormone-sensitive lipase (HSL) and adipose triglyceride lipase (ATGL) to FA and glycerol, for use by other tissues in ATP generation and/or hepatic gluconeogenesis. CNTF treatment for 24 or 48 h resulted in a significantly reduced expression of FAS mRNA that was matched by a significant reduction in SREBP-1 transcript (Figure 4A). Moreover, HSL mRNA increased significantly after CNTF treatment for 24 h and ATGL mRNA increased significantly after treatment for 48 h (Figure 4B). Importantly, Western blotting experiments performed on hMADS adipocyte protein extracts confirmed the reduced expression of FAS protein (Figure 4C) and the increased expression of ATGL protein (Figure 4D) following CNTF treatment for 24 and 48 h and CNTF treatment for 48 h induced a significant increase of FFA release into the culture media (Supplementary Figure 1). Next, evaluation of the expression of PPARγ coactivator (PGC)-1α, carnitine palmitoyltransferase 1 (CPT1), mitochondrial transcription factor A (TFAM), nuclear respiratory factor 1 (NRF1), and UCP1 showed that CNTF treatment for 24 and 48 h increased the expression of PGC-1α mRNA and protein (Figure 4E) and of CPT1 mRNA (Figure 4F), whereas the expression of TFAM, NRF1, and UCP1 was not affected (data not shown). PGC-1α is the master regulator of mitochondrial biogenesis in adipocytes (41), and CPT1 is an essential step in long-chain FA beta oxidation (42). Taken together, these data suggest that long-term CNTF treatment inhibits lipid synthesis, stimulates FA breakdown, induces mitochondrial biogenesis, and promotes lipid oxidation in hMADS adipocytes, without enhancing their capacity for uncoupled respiration.

### CNTF Blunts Inflammatory Gene Expression and Ameliorates Insulin Resistance in TNFα-treated hMADS Adipocytes

**Figure 5:**
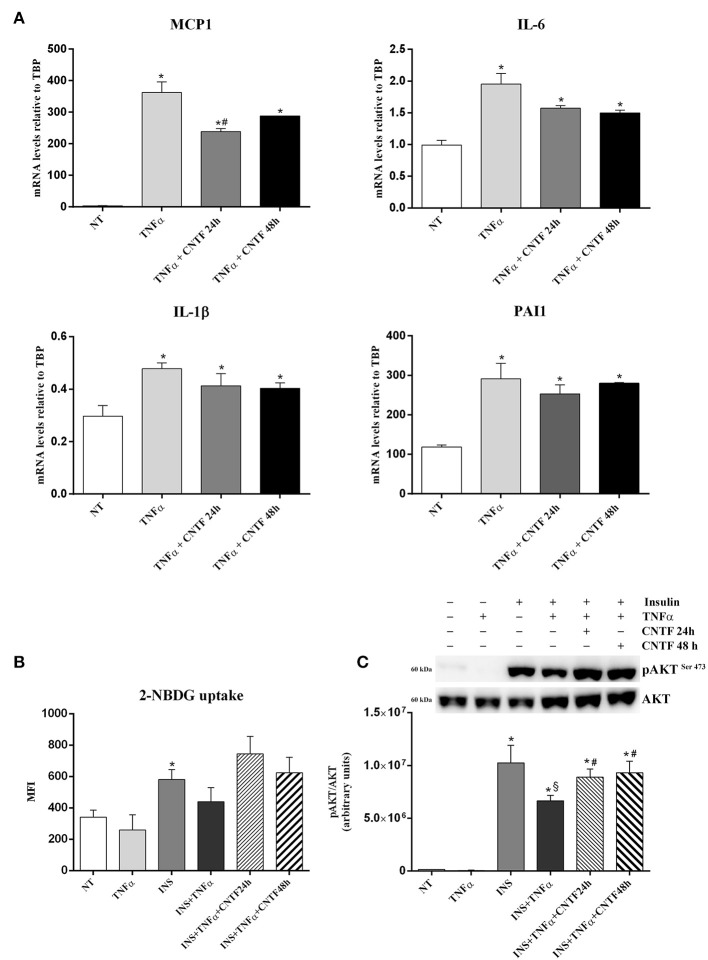
Anti-inflammatory effects of CNTF in hMADS adipocytes. **(A)** MCP1, IL-6, IL-1β, and PAI1 mRNA levels in hMADS adipocytes treated for 24 h with 10 ng/ml TNFα alone or administered with 1 nM CNTF for 24 or 48 h. Data (*n* = 3) are mean ± SEM, *p < 0.05 compared with untreated cells (NT), #*p* < 0.05 compared with cells treated with TNFα. Data were analyzed using one-way ANOVA. 2-NBDG uptake, expressed as mean fluorescent intensity (MFI), **(B)** and representative immunoblot and quantification of 473-serine AKT phosphorylation **(C)** in hMADS adipocytes treated with 10 ng/ml TNFα for 24 h; with 100 nM insulin for 20 min; with TNFα and insulin; or with TNFα, insulin and 1 nM CNTF for 24 or 48 h. Data (*n* = 3) are mean ± SEM, *p < 0.05 compared with untreated cells (NT), $*p* < 0.05 compared with cells treated with insulin, #*p* < 0.05 compared with cells treated with insulin and TNFα. Data were analyzed using one-way ANOVA.

In obese fat, chronic lipid, and carbohydrate overload results in adipocyte hypertrophy, stress, and death; stressed and dead adipocytes induce a state of tissue inflammation that is characterized by adipokine dysregulation, fibrosis, and infiltration by inflammatory cells, mainly macrophages (19, 20, 43–45). These phenomena are accompanied by abnormal release of FA, adipokines, and proinflammatory molecules including TNFα, interleukin (IL)-6, and IL-1β which, by acting on target organs, induce insulin resistance and favor the onset of type 2 diabetes (21, 46). To establish whether CNTF exerts a beneficial role on obese fat, we stressed hMADS adipocytes with 10 ng/ml TNFα for 24 h (47, 48) and evaluated inflammatory cytokine expression in response to CNTF treatment. As expected, TNFα treatment was accompanied by a significantly increased expression of monocyte chemoattractant protein 1 (MCP-1), IL-6, IL-1β, and plasminogen activator inhibitor 1 (PAI1) (Figure 5A). Interestingly, pretreatment with CNTF for 48 h and co-treatment for 24 h significantly blunted the increased expression of MCP1 mRNA induced by TNFα (Figure 5A); IL-6, IL-1β, and PAI1 levels diminished, but not significantly. TNFα treatment of cultured adipocytes also results in a variable level of insulin resistance (47). Indeed, TNFα-treated hMADS cells showed a reduced, albeit not significant, insulin-dependent glucose uptake that was partially restored by treatment with CNTF for 24 or 48 h (Figure 5B). Interestingly, in these experimental conditions CNTF was able to counteract the reduction in insulin-induced AKT phosphorylation seen in TNFα-treated hMADS (Figure 5C).

## Discussion

CNTF belongs to the glycoprotein 130 (gp130) cytokine family along with IL-6, leukemia inhibitory factor (LIF), oncostatin M (OSM), and cardiotrophin-1. Binding of each member of this family to its specific receptor α is followed by activation of common receptor subunit gp130. By participating in the modulation of several intracellular pathways, gp130 cytokines induce pleiotropic, redundant and partially overlapping effects on a variety of biological processes including hematopoiesis, neuronal differentiation, bone remodeling and inflammation. Several studies have also stressed their role in body metabolism and lipid and glucose homeostasis (49). Among them, CNTF is the cytokine attracting the strongest interest, due to its anti-obesity effect in animal models (6, 7, 10) as well as in leptin-resistant obese patients (5).

Here, we describe for the first time some cellular effects of CNTF on hMADS adipocytes, a well-established human white adipocyte model (26–28). As in mouse 3T3-L1 cells undergoing adipose differentiation (24), CNTFRα protein expression is downregulated even during hMADS differentiation. Interestingly, differentiated hMADS adipocytes express higher levels of CNTFRα mRNA than undifferentiated hMADS cells. Even whether a positive correlation between the levels of protein and the corresponding mRNA is generally expected in eukaryotic cells, low protein expression in the presence of high mRNA levels is becoming a quite frequent observation, possibly due to complicated and varied post-transcriptional mechanisms involved in turning mRNA into protein, regulation of mRNA by specific microRNAs, and post-transduction management of proteins due to protein half-life (for example, rate of proteasomal degradation and proteolytic cleavage) (50–52). At present, the possible mechanisms involved in the mismatch between CNTFRα mRNA and protein levels occurring in hMADS cells and adipocytes are unknown. The high expression of CNTFR protein in hMADS cells suggests that, like LIF (53) and OSM (54), CNTF may play a role in preadipocyte proliferation and/or differentiation. Importantly, whereas fully differentiated 3T3-L1 adipocytes do not express CNTFRα (24), fully differentiated hMADS adipocytes retain detectable CNTFRα levels. This makes hMADS adipocytes a highly useful model to study the metabolic action of CNTF and dissect its molecular mechanisms at the cell level.

In hMADS adipocytes, CNTF readily and consistently activated the JAK-STAT3 pathway, as reported in other cellular targets including neurons (7, 9), ependymal cells and tanycytes (55, 56), skeletal muscle fibers (11), and pancreatic cells (16, 17). Moreover, the acute and transient activation of the AMPK and AKT pathways induced in hMADS adipocytes suggests that CNTF exerts a complex action on several adipocyte signaling systems. However, the persistence among differentiated hMADS adipocytes of undifferentiated cells expressing high CNTFRα levels due to the nature of the cell line (26, 27), raises the question of whether and to what extent signaling activation is also due to stimulation of these cells.

CNTF treatment for 24 and 48 h reduced FAS and SREBP-1 expression in hMADS adipocytes, possibly reflecting a reduced FA synthesis, and increased the expression of the lipolytic enzymes HSL and ATGL. Interestingly, such genetic modifications were accompanied by upregulation of the mitochondrial markers PGC-1α and CPT1. These findings are consistent with the transcriptional changes described in 3T3-L1 adipocytes (24, 25). Collectively, these data suggest that also in humans CNTF treatment has the potential to induce adipocyte mitochondriogenesis, lipolysis, and FA oxidation.

hMADS adipocytes also exhibited a significant increase in basal glucose uptake induced by 20 min CNTF treatment. This acute effect was concomitant with the activation of AKT signaling, which in mature adipocytes is closely related to glucose uptake through GLUT4 translocation (38). At the same time point, CNTF also promoted insulin-dependent AKT signaling phosphorylation. Longer-term CNTF treatment promoted insulin-dependent AKT activation, but not to a significant extent. Similarly, insulin-dependent glucose uptake increased, albeit not significantly, in these conditions. Collectively, these data suggest that in hMADS adipocytes CNTF may, to some extent, affect both basal and insulin-dependent glucose entry, possibly through the AKT signaling. Notably, in skeletal muscle fibers (11, 12) and hepatocytes (14) CNTF enhances responsiveness to insulin, whereas in 3T3-L1 adipocytes it increases the expression of GLUT4 and insulin-induced insulin receptor substrate-1 (24).

There is a close link between obesity, chronic inflammation and insulin resistance, the degree of fat inflammation being closely dependent on the number of macrophages infiltrating obese fat (19, 20, 43–45). In TNFα-treated hMADS adipocytes, taken as an *in vitro* model of adipose tissue inflammation (47, 48), CNTF significantly reduced the expression of MCP1, the main chemotactic factor, which leads to infiltration of inflammatory cells in obese fat (57). This finding indicates that CNTF may have anti-inflammatory properties. Moreover, CNTF significantly reduced TNFα-induced AKT inhibition and showed a tendency to promote concomitant insulin-dependent glucose uptake. Collectively, these data lend support to a possible role of CNTF as an insulin-sensitizer, which could be harnessed to overcome obesity-related adipose inflammation and insulin resistance.

Our study did not inquire into which signaling pathway(s) specifically induce(s) the distinctive cellular effects exerted by CNTF. Further work using selective transduction pathway agonists and antagonists is required to characterize the upstream molecular events leading to CNTF-induced transcriptional or metabolic effects on adipocytes. Since mice lacking STAT3 in adipose tissue exhibit increased body weight, adipocyte hypertrophy and reduced lipolysis (58), JAK-STAT3 signaling could explain at least some transcriptional changes induced in hMADS adipocytes. As in rodent skeletal muscle (11) and fat (25), the promotion of fat oxidation by CNTF in hMADS adipocytes may be related to the AMPK pathway which, however, also exerts an anti-inflammatory action on obese adipocytes (36), and may be involved in the CNTF-dependent MCP-1 downregulation we described in TNFα-treated adipocytes.

In conclusion, our data show that the ability of CNTF to convert adipocytes to a less lipid-storing and a more energy expenditure-prone phenotype is conserved in human cells. A greater understanding of its biological actions in human adipocytes is expected to provide useful information for the development of novel therapeutic strategies against obesity and related diseases.

## Data Availability Statement

All datasets generated for this study are included in the article/Supplementary Material.

## Author Contributions

JP: conception and design, performance of experiments, data analysis and interpretation, manuscript writing. ED, GT, IS, FM, MR and MT: performance of experiments and data analysis and interpretation. CD: critical revision of the manuscript. SC: financial support and critical revision of the manuscript. AG: conception and design, financial support, data analysis and interpretation, manuscript writing and final approval of the manuscript. All authors approved the final version of the manuscript.

### Conflict of Interest

The authors declare that the research was conducted in the absence of any commercial or financial relationships that could be construed as a potential conflict of interest.

## Supplementary Material

Supplementary Figure 1 Lipolysis induced by long-term CNTF treatment in hMADS adipocytes. Lipolysis was assessed through FFA release into the culture media of hMADS adipocytes treated with 1 nM CNTF for 24 and 48 h and 0.5 mM IBMX for 24 h (positive control). Results were expressed as fold changes. Data (*n* = 3) are mean ± SEM, **p* < 0.05 compared with untreated cells (NT). Data were analyzed using one-way ANOVA.
