# Supplementary figures and images for: Biological Effects of Ciliary Neurotrophic Factor on hMADS Adipocytes
Source: Front Endocrinol (Lausanne). 2019 Nov 12;10:768. doi: 10.3389/fendo.2019.00768 (PMC6861295; doi:10.3389/fendo.2019.00768)

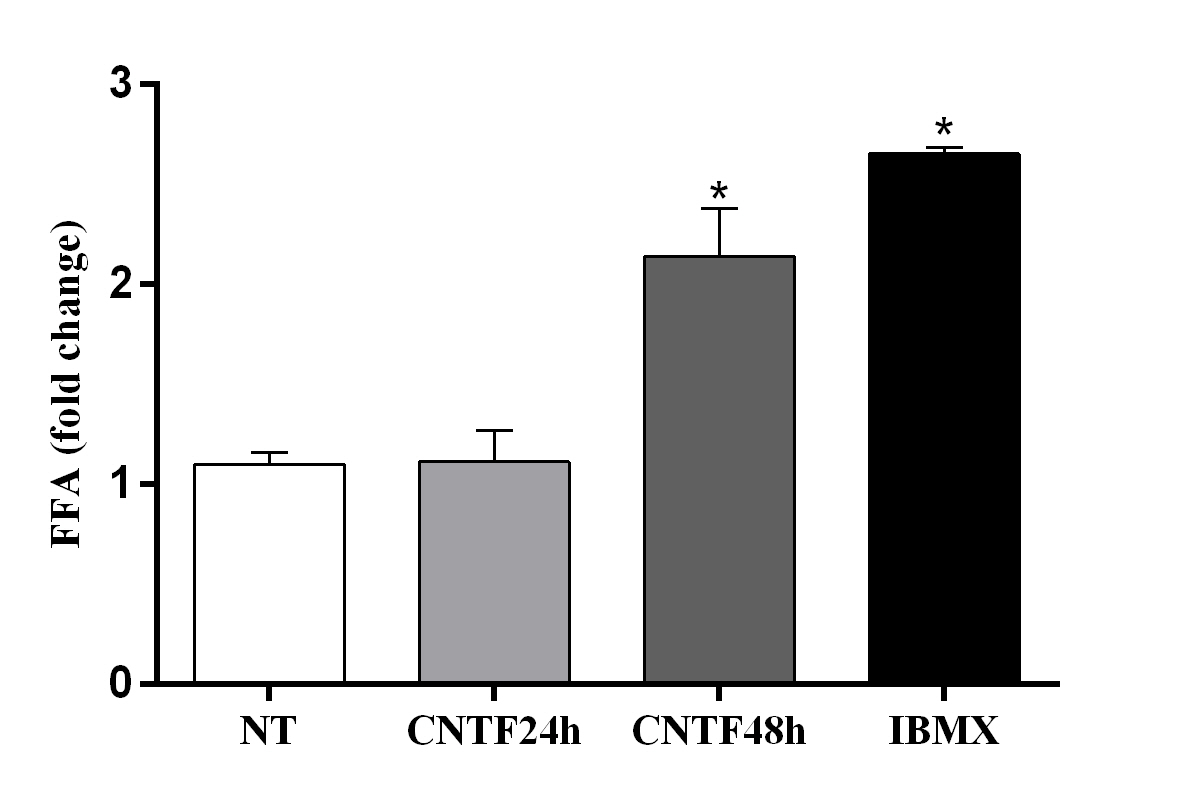

Supplement: Supplementary Figure 1 — Lipolysis induced by long-term CNTF treatment in hMADS adipocytes. Lipolysis was assessed through FFA release into the culture media of hMADS adipocytes treated with 1 nM CNTF for 24 and 48 h and 0.5 mM IBMX for 24 h (positive control). Results were expressed as fold changes. Data (n = 3) are mean ± SEM, *p < 0.05 compared with untreated cells (NT). Data were analyzed using one-way ANOVA. [file Image_1.JPEG]
